# Human-AI interaction in skin cancer diagnosis: a systematic review and meta-analysis

**DOI:** 10.1038/s41746-024-01031-w

**Published:** 2024-04-09

**Authors:** Isabelle Krakowski, Jiyeong Kim, Zhuo Ran Cai, Roxana Daneshjou, Jan Lapins, Hanna Eriksson, Anastasia Lykou, Eleni Linos

**Affiliations:** 1 Center for Digital Health, Stanford University School of Medicine, Stanford, CA USA; 2 Department of Oncology and Pathology, Karolinska Institutet, Stockholm, Sweden; 3 Department of Dermatology, Stanford, Stanford University, Stanford, CA USA; 4 Department of Dermatology, Department of Biomedical Data Science, Stanford School of Medicine, Stanford, CA USA; 5 Department of Dermatology, Theme Inflammation, Karolinska University Hospital, Stockholm, Sweden; 6 Theme Cancer, Unit of Head-Neck-, Lung- and Skin Cancer, Skin Cancer Center, Karolinska University Hospital, Stockholm, Sweden; 7 Department of Education, University of Nicosia, Nicosia, Cyprus

**Keywords:** Skin cancer, Skin manifestations, Diagnosis

## Abstract

The development of diagnostic tools for skin cancer based on artificial intelligence (AI) is increasing rapidly and will likely soon be widely implemented in clinical use. Even though the performance of these algorithms is promising in theory, there is limited evidence on the impact of AI assistance on human diagnostic decisions. Therefore, the aim of this systematic review and meta-analysis was to study the effect of AI assistance on the accuracy of skin cancer diagnosis. We searched PubMed, Embase, IEE Xplore, Scopus and conference proceedings for articles from 1/1/2017 to 11/8/2022. We included studies comparing the performance of clinicians diagnosing at least one skin cancer with and without deep learning-based AI assistance. Summary estimates of sensitivity and specificity of diagnostic accuracy with versus without AI assistance were computed using a bivariate random effects model. We identified 2983 studies, of which ten were eligible for meta-analysis. For clinicians without AI assistance, pooled sensitivity was 74.8% (95% CI 68.6–80.1) and specificity was 81.5% (95% CI 73.9–87.3). For AI-assisted clinicians, the overall sensitivity was 81.1% (95% CI 74.4–86.5) and specificity was 86.1% (95% CI 79.2–90.9). AI benefitted medical professionals of all experience levels in subgroup analyses, with the largest improvement among non-dermatologists. No publication bias was detected, and sensitivity analysis revealed that the findings were robust. AI in the hands of clinicians has the potential to improve diagnostic accuracy in skin cancer diagnosis. Given that most studies were conducted in experimental settings, we encourage future studies to further investigate these potential benefits in real-life settings.

## Introduction

As a result of increasing data availability and computational power, artificial intelligence (AI) algorithms—have reached a level of sophistication that enables them to take on complex tasks previously only conducted by human beings^1^. Several AI algorithms are now approved by the United States Food and Drug Administration (FDA) for medical use^2–4^. Though there are currently no image-based dermatology AI applications that have FDA approval, several are in development^2^.

Skin cancer diagnosis relies heavily on the interpretation of visual patterns, making it a complex task that requires extensive training in dermatology and dermatoscopy^5,6^. However, AI algorithms have been shown to accurately diagnose skin cancers, even outperforming experienced dermatologists in image classification tasks in constrained settings^7–9^. However, these algorithms can be sensitive to data distribution shifts. Therefore, AI-human partnerships could provide performance improvements that surmount the limitations of both human clinicians or AI alone. Notably, Tschandl et al. demonstrated in their 2020 paper that the accuracy of clinicians supported by AI algorithms surpassed that of either clinicians or AI algorithms working separately^10^. This approach of an AI-clinician partnership is considered the most likely clinical use of AI in dermatology, given the ethical and legal concerns of automated diagnosis alone. Therefore, there is an urgent need to better understand how the use of AI by clinicians affects decision making^11^. The goal of this study was to evaluate the diagnostic accuracy of clinicians with vs. without AI assistance using a systematic review and meta-analysis of the available literature.

## Results

### Literature search and screening

**Fig. 1:**
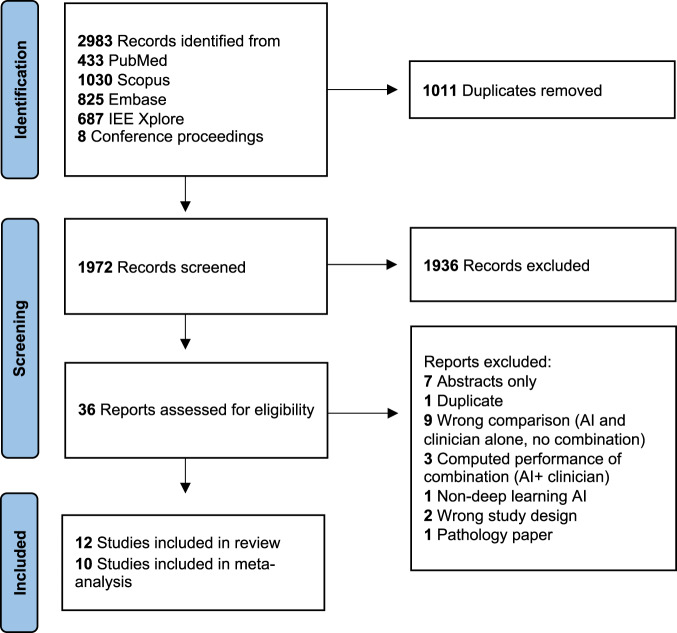
Study selection. Flow diagram of the study selection process.

For this systematic review and meta-analysis, 2983 records were initially retrieved, of which, 1972 abstracts were screened after the automatic duplicate removal by Covidence (Fig. 1). As 1936 articles were considered irrelevant and further excluded, the full text of 36 articles was reviewed. A total of 12 studies were included in the systematic review^10,12–22^ and ten studies were included in the meta-analysis^10,12–15,17,19–22^, whereas the information needed to create contingency tables of AI-assisted and un-assisted medical professionals was unavailable in two studies^16,18^.

### Study characteristics

Tables 1 and 2 presents the characteristics of the included studies. Half of the studies were conducted in Asia (50%, South Korea=5, China=1) and the other half was done in North/South America (25%, USA = 1, Argentina=1, Chile=1), and Europe (25%, Austria=1, Germany=1, Switzerland=1). More studies were performed in experimental (67%, *n* = 8) than clinical settings (33% *n* = 4). A quarter of studies included only dermatologists (25%, *n* = 3), more than a half (58%, *n* = 7) included a combination of dermatology specialists (e.g., dermatologist and dermatology residents) and non-dermatology medical professionals (e.g., primary care physicians, nurse practitioners, medical students) and among these, two studies included lay persons, but this data was not included for meta-analysis. In two studies (17%), only non-dermatology medical professionals were included. The median number of study participants was 18.5, ranging from 7 to 302.

**Table 1:** Study characteristics of the 12 included studies

Source	Target condition	Setting and design	Participants	Patients	Reference standard	Classification task	No. of cases/ participant
Ba et al.^21^	Cutaneous tumors (10 categories: BCC, SCC, MM, Bowen disease, AK, melanocytic nevus, SK, haemangioma, DF, wart)	Reader study, China	18 dermatologists from 9 different hospitals	400 randomly selected cases from the test set (40 images per category). Clinical images only. No clinical information.	Histopathology on all images	Classification of clinical images without and with AI assistance, (washout period of 2 months).	400
Cho et al.^21^	Benign (LP, melanotic macule, pyogenic granuloma, plasma cell cheilitis and venous lake) vs. malignant lip disease (SCC, BCC, MM, adenocarcinoma, actinic cheilitis, Bowen disease, MMis)	Reader study, Korea	44 physicians and medical students (6 dermatologists, 12 dermatology residents, 9 non-dermatology specialists, 17 medical students)	40 randomly chosen cases (20 benign, 20 malignant). Clinical images only.	Histopathology or clinical diagnosis	Rate images as benign or malignant, first without AI assistance and then with.	40
Han et al.^a^^,14^	Malignant vs. benign skin lesion + treatment recommendation (malignant nodule, benign lesions requiring antifungals, antivirals, antibiotics, or steroid treatment, and other benign lesions)	Reader study, Korea	47 physicians (21 dermatologists, 26 dermatology residents) and 23 non-medical professionals	240 randomly selected clinical images without clinical data from the SNU dataset	Histopathology, or clinical diagnosis	Classification of clinical images as malignant or benign, with treatment suggestion. Next, AI assistance and new classification.	240
Han et al.^19^	Skin lesions suspicious for skin cancer	Prospective clinical study, Korea	4 dermatology residents and 4 non-dermatology trainees.4 dermatologists served as ground truth for un-biopsied lesions (not exposed to AI).	576 patients with suspected skin cancer (by either patient or physician) Randomization to standard clinical in-person examination without AI (*n* = 281) or with AI (*n* = 295).	Histopathology, or clinical diagnosis	If randomization to AI: patient examination, top 3 diagnoses, then AI assistance and a possibility to modify initial diagnoses. Control group: patient examination, top 3 diagnoses without AI. No dermoscopy was allowed for any study participants.	20-53/participant
Jahn et al.^17^	Nevus vs. MM	Prospective clinical study, Switzerland	7 dermatologists	114 patients (1204 lesions) with a high risk for developing a MM. In-person examination with dermoscopy.	Histopathology, or study participant in concordance with scores of the two AI tools^b^	Assessment by dermatologist, then all melanocytic nevi ≥3 mm or any smaller suspicious lesions were assessed by the three AI tools. Dermatologist was informed of the results of the MoleAnalyzer Pro + Dexi and could modify the initial diagnosis.	N/A
Jain et al.^15^	Different skin conditions, including skin cancer (120 different diagnoses)	Reader study, USA	40 medical professionals (20 primary care physicians and 20 nurse practitioners)	1048 cases from two retrospective datasets from California and Hawaii, used for validation of the algorithm. Clinical images and clinical information.	Histopathology or consensus^b^	Provide top diagnoses. Participants were divided into two groups with the same cases but opposite assistance modalities (ie, unassisted vs. AI assisted) for each case.	1048 (half with and the rest without AI assistance)
Kim et al.^20^	Skin lesions suspicious for skin cancer	Prospective clinical study, Korea	11 dermatology residents, 7 interns. 10 dermatologists served as ground truth for un-biopsied lesions, (not exposed to AI).	285 patients with suspected skin cancer (by either patient or physician) were randomized to standard clinical in-person examination without AI (*n* = 141 patients) or with AI (*n* = 144 patients).	Histopathology or clinical diagnosis	If randomization to AI: patient examination, top three diagnoses. Then AI assistance and a possibility to modify initial diagnoses.Control group: patient examination, top 3 diagnoses without AI.	N/A
Lee et al.^12^	Nevus vs. acral MM	Reader study, Korea	60 physicians (20 dermatologists, 20 dermatology residents, 20 primary care physicians)	100 cases from the test-set. Dermoscopic images and clinical information.	Histopathology or consensus (3 dermatologists and follow-up)	Classification of 100 dermoscopic images. After two weeks same images + clinical info. After another two weeks same images + clinical info + AI assistance. Rate diagnosis + management.	100
Lucius et al.^18^	Pigmented skin lesions (7 categories: melanocytic nevi, vascular skin lesions, benign keratoses, DF, intraepithelial carcinomas, BCC, and MM)	Reader study, Argentina	19 primary care physicians	70 dermoscopic images from the HAM 10,000 dataset. Clinical data: N/A	Histopathology, biology (>1.5 years of sequential dermoscopic imaging without changes), expert consensus and in vivo confocal images	Classification of 35 dermoscopic images without algorithmic assistance, then 35 new images with algorithmic assistance. Time constraint: 45 seconds.	70
Maron et al.^22^	MM vs. nevus	Reader study, Germany	12 dermatologists from 9 university hospitals	1200 dermoscopic images from the ISIC archive. No clinical data.	Histopathology on all images	First assessment without AI assistance. Next, the same images with dermatologist’s old assessment + AI assistance and possibility to modify diagnosis. Diagnosis, image quality rating and confidence was recorded. Participants were informed of the classifier’s performance.	100 (12 individual test sets)
Muñoz-López et al.^c^^,16^	Skin lesions (5 categories: inflammatory, infectious, neoplastic, alopecia, other) with 13 subcategories.	Prospective clinical study, Chile	Unknown number of dermatologists participated in the telemedicine study. (9 medical professionals participated in a reader study diagnosing the lesions without the help of AI)	281 consecutive patients submitting clinical images of 340 skin lesions (taken by the patient) and sharing clinical information during telemedicine visit.	Histopathology or clinical diagnosis, otherwise consensus of 6 dermatologists	Dermatologist reviewed clinical images + interviewed patients on a video call. Recorded diagnosis + treatment plan + uploaded image to AI tool (could impact the outcome). Recorded if AI was considered helpful + if it changed initial diagnosis (no before and after diagnosis was recorded).	N/A
Tschandl et al.^a^^,10^	Pigmented skin lesions (7 categories: MM, BCC, AK + intraepithelial carcinomas, nevi, benign keratinocytic lesions, DF and vascular lesions)	Reader study, Austria (study participants from 41 countries)	302 medical professionals (169 dermatologists 77 dermatology residents, 38 primary care physicians, 4 medical specialists, 1 medical student, 4 non-medical, 7 nurse practitioners, 2 residents) from 41 countries	1412 of 1511 Dermoscopic images from the ISIC 2018 challenge without clinical data.	Histopathology, biology (>1.5 years of sequential dermoscopic imaging without changes), expert consensus, or in vivo confocal images	Classification of batches of 28 dermoscopic images first without, then with one type of AI support, or with crowd-based decision support. Possibility to modify diagnosis. Time used as a surrogate for confidence.	One test = 28 images, each rater could perform up to five tests

*BCC* basal cell carcinoma, *SCC* squamous cell carcinoma, *DF* dermatofibroma, *SK* seborrheic keratosis, *AK* actinic keratosis, *MM* malignant melanoma, *MMis* melanoma in situ, *LP* lichen planus, *N/A* not applicable, *HAM* 10,000: Human Against Machine with 10,000 training images dataset, *ISIC* International Skin Imaging Collaboration, *SNU* Seoul National University Bundang Hospital, Inje University Sanggye Paik Hospital, and Hallym University Dongtan Hospital, clinical diagnosis: the diagnosis made by the treating dermatologist

^a^More than one experiment was performed in the study, but only the experiment that was included in the meta-analysis is reported in the table.

^b^Only histologically confirmed cases were included in the meta-analysis.

^c^The effect of the AI assistance was not evaluated with a before and after diagnosis, only self-reported.

**Table 2:** Characteristics of the algorithms and data used in the included studies

Source	Output of algorithm	Algorithm	No. images for training	Data source	External validation?	Private sector funding?
Ba et al.^21^	Top 3 diagnoses	AI skinreader (EfficientNet-B3)	25,773 images training and validation (80/20 split)	Chinese PLA General Hospital & Medical School	No	No
Cho et al.^13^	Malignancy probability (0–100%)	Inception-Resnet-V2	1629 images	Seoul National University Hospital, Seoul National University Bundang Hospital and SMG-SNU Boramae Medical Center	Yes (dataset from other hospitals)	No
Han et al.^14^	Top 3 diagnoses, binary malignancy prediction (benign vs malignant), and treatment prediction (steroids vs antibiotics vs antivirals vs antifungals)	Model Dermatology build 2018 (SENet, SE-ResNet-50, VGG-19)	220,680 images	ASAN, Web, MED-NODE^a^, Normal, Edinburgh and SNU	Yes (datasets from other hospitals)	No
Han et al.^19^	Top 5 diagnoses and malignancy score (0–100)	Model Dermatology build 2020 (SENet, SE-ResNeXt-101, SE-ResNeXt-50, ResNeSt-101, and ResNeSt-50)	4,204,323 images crops [unknown number of images]	Han et al. 2020 + Seven-Point Checklist Dermatology Dataset^a^ and semi-automatically annotated images on the internet	Yes (prospective dermatology cases in Korea)	No
Jahn et al.^17^	MoleAnalyzer Pro: malignancy score 0–1.0 (threshold >0.5) DEXI: malignancy score 0.0–10.0 (threshold >5.0)	FotoFinder ATBM® with MoleAnalyzer Pro (Inception-v4) + 3D TBP Vectra® WB360 system with DEXI. (SkinVision® was evaluated but not used for decision making)	N/A	MoleAnalyzer Pro: ISIC^a^ + cooperating dermatologists DEXI: N/A	N/A	No
Jain et al.^15^	Up to 5 of the AI’s top predictions, confidence level (0-5) and similar images	Inception-v4	64,837 images	Retrospective consecutive adult cases from a teledermatology service serving 17 primary-care and specialist sites from two states in the United States.	Yes (dataset from different time period)	Yes
Kim et al.^20^	Top 3 diagnoses and malignancy score (0–100)	Model Dermatology build 2019 (SE-Net, SE-ResNeXt-50)	721,749 image crops [unknown number of images]	Han et al. 2020 + Seven-Point Checklist Dermatology Dataset^a^ and semi-automatically annotated images on the internet	Yes (prospective dermatology cases in Korea)	No
Lee et al.^12^	Nevus vs. acral MM and probability (0–100%)	ALMnet (ResNet 50)	872 images	Department of Dermatology, Severance Hospital, Seoul, Korea	No	Yes, part of the funding
Lucius et al.^18^	Top diagnosis	EfficientNetB5	8313 images	HAM 10,000^a^	No	No
Maron et al.^22^	Nevus vs. MM and probability (0–100%)	ResNet 50	4894 images	ISIC (with a large fraction from the HAM 10,000 dataset)^a^	No	No
Muñoz-López et al.^16^	Top 3 diagnoses	Model Dermatology build 2018 (SENet, SE-ResNet-50, VGG-19) (Same as Han et al. 2020)	220,680 images	ASAN, Web, MED-NODE^a^Normal, Edinburgh and SNU (Same as Han et al. 2020)	Yes (prospective teledermatology cases in Chile)	No
Tschandl et al.^10^	Either multiclass probabilities, probability of malignancy or AI-based CBIR (images of similar lesions)	ResNet34	8012 images	HAM 10,000^a^	No	No

*N/A* not applicable, *HAM* 10,000 Human Against Machine with 10,000 training images dataset, *ISIC* International Skin Imaging Collaboration, *ASAN* Department of Dermatology at Asan Medical Center, *Web* images obtained from the internet, *MED-NODE* Department of Dermatology at the University Medical Center Groningen, and Normal: images of skin with normal or nonspecific findings, Edinburgh: Dermofit Image Library from the University of Edinburgh, *SNU* Seoul National University Bundang Hospital, Inje University Sanggye Paik Hospital, and Hallym University Dongtan Hospital, *CBIR* Content-Based Image Retrieval.

^a^Open-source dataset.

Clinical information was provided to study participants in addition to images or in-patient visits in half of the studies (50%, *n* = 6). For diagnosis, outpatient clinical images were most frequently provided (42%, *n* = 5), followed by dermoscopic images (33%, *n* = 4) and in-patient visits (25%, *n* = 3). Diagnostic task was either choosing the most likely diagnosis (58%, *n* = 7) or rating the lesion as malignant vs. benign (42%, *n* = 5). Most studies (75%, *n* = 9) used a paired design with the same reader diagnosing the same case first without, then with AI assistance, whereas two studies provided different images between the two tasks. A fully crossed design (i.e., all readers diagnosing all cases in both modalities) was performed in four studies. One study only reported diagnosis with AI support, thus did not allow to analyze the effect of AI^16^. Studies included a reference standard that was either varying combinations of either histopathology, a dermatologist panel’s diagnosis or the treating physician, from medical records, clinical follow-up or in vivo confocal microscopy (75%, *n* = 9) or histopathologic diagnosis on all images (17%, *n* = 2). One study considered either histopathology or the study participant being in concordance with two AI tools that were studied as the reference standard^17^. Most AI algorithms did not provide explanation for their outputs or presentation beyond the top-1 or top-3 diagnoses along with their respective probabilities or a binary malignancy score. Content-based image retrieval (CBIR) was the only explainability method that was used, namely in two of the studies (17%) and Tschandl et al.^10^ was the only study that delved into the effects of various representation of AI output on the diagnostic performance of physicians. Definition of target condition varied across studies, but all studies included at least one skin cancer among the differential diagnoses. The summary of methodological quality assessments can be found in Supplementary Table 1. Although κ was low (κ = 0.33), the Bowker’s Test of Symmetry^23^ was not significant, hence two raters were considered having the same propensity to select categories. All three assessors agreed with the final quality assessments.

### Meta-analyses results

**Fig. 2:**
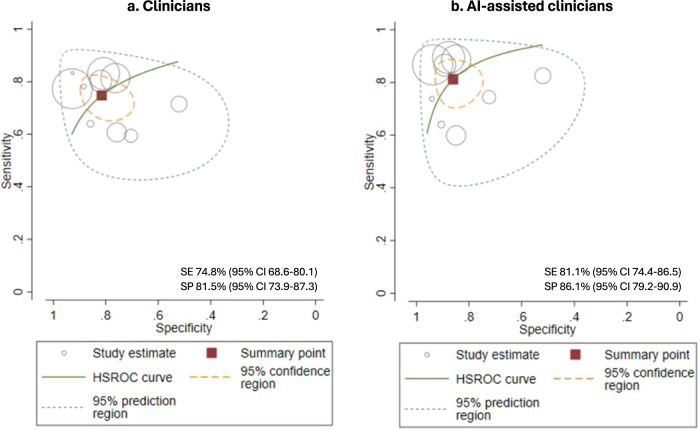
SROC Curves. SE sensitivity, SP specificity. Performance of clinicians with no AI assistance (**a**) compared to AI-assisted clinicians (**b**) in the included studies.

**Fig. 3:**
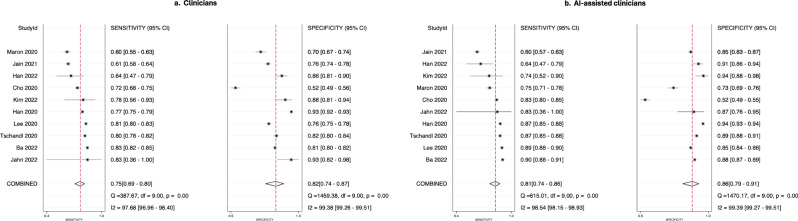
Flow diagram of the study selection process. Forest plots. Meta-analysis results of the diagnostic performance of clinicians without (**a**) or with (**b**) AI assistance.

The summary estimate of sensitivity for clinicians overall was 74.8% (95% CI 68.6–80.1) and specificity 81.5% (73.9–87.3). The overall diagnostic accuracy increased with AI assistance to a pooled sensitivity and specificity of 81.1% (74.4–86.5) and 86.1% (79.2–90.9), respectively. The SROC curves and forest plots of ten studies for clinicians without vs. with AI assistance each are shown in Figs. 2 and 3, respectively, where less heterogeneity is observed in the sensitivity of clinicians overall compared to clinicians with AI assistance.

To investigate the effect of AI assistance in more detail, we conducted subgroup analyses based on clinical experience level, test task and image type (Table 3). We observed that dermatologists had the highest diagnostic accuracy in terms of sensitivity and specificity. Residents (including dermatology residents and interns) were the second most accurate group, followed by non-dermatologists (including primary care providers, nurse practitioners and medical students). Notably, AI assistance significantly improved the sensitivity and specificity of all groups of clinicians. The non-dermatologist group appeared to benefit the most from AI assistance regarding improvement of pooled sensitivity (+13 points) and specificity (+11 points). For classification task, the sensitivity of both binary classification (malignant vs. benign) and top diagnosis improved with AI assistance. Meanwhile, AI assistance significantly improved pooled specificity only for top classification, reaching a specificity of 88.8%, (86.5–90.8). No significant difference was observed for image type.

**Table 3:** Subgroup analysis by clinician’s experience level, image type and classification task

Subgroups (N)	Clinicians (95% CI)		AI-assisted clinicians (95% CI)		*p*-value^c^
Dermatologists	SE	81.8% (76.1–86.4)	SE	86.5% (81.9–90.0)	0.007
(*N* = 7)	SP	79.2% (70.6–85.7)	SP	87.2% (81.1–91.5)	<0.001
Residents^a^	SE	77.8% (71.7–83.0)	SE	85.4% (80.7–89.1)	0.003
(*N* = 6)	SP	80.1% (70.2–87.3)	SP	87.6% (80.4–92.4)	0.007
Other medical professionals^b^	SE	66.3% (55.8–75.3)	SE	79.3% (71.0–85.7)	0.003
(*N* = 4)	SP	70.1% (55.9–81.3)	SP	80.9% (69.4–88.7)	0.011
Binary malignancy prediction	SE	73.0% (63.8–80.6)	SE	84.2% (77.6–89.1)	0.027
(*N* = 5)	SP	78.4% (63.8–88.2)	SP	79.6% (65.5–88.9)	0.892
Top diagnosis	SE	73.3% (63.0–81.6)	SE	81.2% (72.8–87.5)	<0.001
(*N* = 5)	SP	81.4% (78.1–84.3)	SP	88.8% (86.5–90.8)	<0.001
Clinical images	SE	72.7% (61.7–81.6)	SE	79.6% (70.1–86.6)	0.300
(*N* = 5)	SP	80.4% (65.8–89.8)	SP	85.3% (73.1–92.6)	0.528
Dermoscopic images	SE	78.2% (67.6–86.1)	SE	84.8% (76.6–90.4)	0.258
(*N* = 4)	SP	79.8% (71.4–86.2)	SP	84.0% (77.0–89.2)	0.380

*N* number of studies, *SE* sensitivity, *SP* specificity, *CI* confidence interval.

^a^Residents including dermatology residents and interns.

^b^Other medical professionals including general practitioners, other medical specialists, nurse practitioners and medical students.

^c^Based on two-sided *z-*test.

There was no evidence of a small-study effect in regression test asymmetry for both humans without (*p* = 0.33) and with AI assistance (*p* = 0.23). Please see Supplementary Fig. 1 for funnel plots. The Spearman correlation test found that the presence of positive threshold effect was low likely for both groups. Sensitivity analyses revealed that excluding outliers slightly increased the pooled sensitivity and specificity in both groups while the pooled sensitivity and specificity mostly remained unchanged when excluding the low-quality study (Supplementary Table 2).

## Discussion

This systematic review and meta-analysis included 12 studies and 67,700 diagnostic evaluations of potential skin cancer by clinicians with and without AI assistance. Our findings highlight the potential of AI-assisted decision-making in skin cancer diagnosis. All clinicians, regardless of their training level, showed improved diagnostic performance when assisted by AI algorithms. The degree of improvement, however, varied across specialties, with dermatologists exhibiting the smallest increase in diagnostic accuracy and non-dermatologists, including primary care providers, demonstrating the largest improvement. These results suggest that AI assistance may be especially beneficial for clinicians without extensive training in dermatology. Given that many dermatological AI devices have recently obtained regulatory approval in Europe, including some CE marked algorithms utilized in the analyzed studies^24,25^, AI assistance may soon be a standard part of a dermatologist’s toolbox. It is therefore important to better understand the interaction between human and AI in clinical decision-making.

While several studies have been conducted to evaluate the dermatologic use of new AI tools, our review of published studies found that most have only compared human clinician performance with that of AI tools, without considering how clinicians interact with these tools. Two of the studies in this systematic review and meta-analysis reported that clinicians perform worse when the AI tool provides incorrect recommendations^10,19^. This finding underscores the importance of accurate and reliable algorithms in ensuring that AI implementation enhances clinical outcomes, and highlights the need for further research to validate AI-assisted decision-making in medical practice. Notably, in a recent study by Barata et al.^26^, the authors demonstrated that a reinforcement learning model that incorporated human preferences outperformed a supervised learning model. Furthermore, it improved the performance of participating dermatologists in terms of both diagnostic accuracy and optimal management decisions of potential skin cancer when compared to either a supervised learning model or no AI assistance at all. Hence, the development of algorithms in collaboration with clinicians appears to be important for optimizing clinical outcomes.

Only two studies explored the impact of one explainability technique (CBIR) on physician’s diagnostic accuracy or perceived usefulness. The real clinical utility of explainability methods needs to be further examined, and current methods should be viewed as tools to interrogate and troubleshoot AI models^27^. Additionally, prior research has shown that human behavioral traits can affect trust and reliance on AI assistance in general^28,29^. For example, a clinician’s perception and confidence in the AI’s performance on a given task may influence whether they decide to incorporate AI advice in their decision^30^. Moreover, research has also shown that the human’s confidence in their decision, the AI’s confidence level, and whether the human and AI agree all influence if the human incorporates the AI’s advice^30^. To ensure that AI assistance supports and improves diagnostic accuracy, future research should investigate how factors such as personality traits^29^, cognitive style^28^ and cognitive biases^31^ affect diagnostic performance in real clinical situations. Such research would help inform the integration of AI into clinical practice.

Our findings suggest that AI assistance may be particularly beneficial for less experienced clinicians, consistent with prior studies of human-AI interaction in radiology^32^. This highlights the potential of AI assistance as an educational tool for non-dermatologists and for improving diagnostic performance in settings such as primary care or for dermatologists in training. In a subgroup analysis, we observed no significant difference between AI-assisted other medical professionals vs. unassisted dermatologists (data not shown). However, this area warrants further research.

Some limitations need to be considered when interpreting the findings. First, among the ten studies that provided sufficient data to conduct meta-analysis, there were differences in design, number and experience level of participants, target condition definition, classification task, and algorithm output and training. Taken together, this heterogeneity implies that direct comparisons should be interpreted carefully. Furthermore, caution is warranted for the interpretation of the subgroup analyses due to the small sample size of the subgroups (up to seven) and the data structure (i.e., repeated measures) since the same participants examined the clinical images both without and with AI assistance in most studies. Given the low number of studies, we refrained from performing further subgroup analyses, such as, comparing specific cancer diagnoses in the subset of articles where these are available. Despite these limitations, our results from this meta-analysis support the notion that AI assistance can yield a positive effect on clinician diagnostic performance. We were able to adjust for potential sources of heterogeneity, including diagnostic task and clinician experience level when comparing the diagnostic accuracy of clinicians with vs. without AI assistance. Moreover, no signs of publication bias and low likelihood of threshold effects were observed. Lastly, the findings were robust such that the pooled sensitivity and specificity nearly stayed the same after excluding outliers or low-quality studies.

Of note, few studies provided participating clinicians with both clinical data and dermoscopic images, which would be available in a real-life clinical situation. Previous research has shown that the use of dermoscopy enables a relative improvement of diagnostic accuracy of melanoma by almost 50% compared to the naked eye^5^. In one of such study, participants were explicitly not allowed to use dermoscopy during the patient examination^19^. Overall, only four studies were conducted in a prospective clinical setting, and three of these could be included for meta-analysis. Thus, most diagnostic ratings in this meta-analysis were made in experimental settings and do not necessarily reflect the decisions made in a clinical real-world situation.

One of the main concerns regarding the accuracy of AI tools rely on the quality of the data it has been trained on^33^. As only three studies used publicly available datasets, evaluation of the data quality is difficult. Furthermore, darker skin tones were underrepresented in the datasets of the included studies, which is a known problem in the field, as most papers do not report skin tone outputs^34^. However, datasets with diverse skin tones have been developed and made publicly available as an effort to reduce disparity in AI performance in dermatology^35,36^. Moreover, few studies provided detailed information about the origin and number of images that had been used for training, validation, and testing of the AI tool and different definitions of these terms were used across studies. There is a need for better transparency guidelines for AI tool reporting to enable users and readers to understand the limits and capabilities of these diagnostic tools. Efforts are being made to develop guidelines that are adapted for this purpose, including the STARD-AI^37^, TRIPOD-AI and, PROBAST-AI^38^ guidelines, as well as the dermatology-specific CLEAR Derm guidelines^39^. In addition, PRISMA-AI^40^ guidelines for systematic reviews and meta-analyses are being developed. These are promising initiatives that will hopefully make both the reporting and evaluation of AI diagnostic tool research more transparent.

## Conclusion

The results of this systematic review and meta-analysis indicate that clinicians benefit from AI assistance in skin cancer diagnosis regardless of their experience level. Clinicians with the least experience in dermatology may benefit the most from AI assistance. Our findings are timely as AI is expected to be widely implemented in clinical work globally in the near future. Notably, only four of the identified studies were conducted in clinical settings, three of which could be included in the meta-analysis. Therefore, there is an urgent need for more prospective clinical studies conducted in real-life settings where AI is intended to be used, in order to better understand and anticipate the effect of AI on clinical decision making.

## Methods

### Search strategy and selection criteria

We searched four electronic databases, including PubMed, Embase, Institute of Electrical and Electronics Engineers Xplore (IEE Xplore) and Scopus for peer-reviewed articles of AI-assisted skin cancer diagnosis without language restriction from January 1, 2017, until November 8, 2022. Search terms were combined for four key concepts: (1) AI, (2) skin cancer, (3) diagnosis, (4) doctors. The full search strategy is available in the Supplementary material (Supplementary Table 3). We chose 2017 as the cutoff for this review since this was the year when deep learning was first reported as performing at a level comparable to dermatologists, notably in the seminal study by Esteva et al^9^, which suggested that AI technology had reached a clinically useful level in assisting skin cancer diagnosis.

We applied Google Translate software for abstract screening of non-English articles. Manual searches were performed for conference proceedings, including NeurIPS, HICSS, ICML, ICLR, AAAI, CVPR, CHIL and ML4Health, and to identify additional relevant articles by reviewing bibliographies and citations of the screened papers and searching Google Scholar.

We included studies comparing diagnostic accuracy of clinicians detecting skin cancer with and without AI assistance. If studies provided diagnostic data from medical professionals other than physicians this data was also included for analysis, as long as the study also included physicians. However, we excluded studies if (1) diagnosis was not made from either images of skin lesions or in-person visits (e.g., pathology slides), (2) diagnostic accuracy was only compared between clinicians and an AI algorithm, (3) non-deep learning techniques were used, or (4) the articles were editorials, reviews, and case reports. We did not limit participants’ expertise, study design or sample size, reference standard, or skin diagnosis if at least one skin malignancy was included in the study. We contacted nine authors to request additional data and clarifications required for the meta-analysis and received data from five of them^10,12–15^ and clarifications from two^16,17^. In four studies^10,14,15,17^ raw data was not available for all experiments or lesions, and our meta-analysis included the data that was available. Studies with insufficient data to construct contingency tables^16,18^ were included in the systematic review but not in the meta-analysis.

Three reviewers performed eligibility assessment, data extraction, and study quality evaluations (IK, JK, ZRC). Commonly used standardized programs were employed for duplicate removal, title and abstract screening, and full-text review (Covidence) and data extraction (Microsoft Excel). Paired reviewers independently screened the titles and abstracts using predefined criteria and extracted data. Disagreement was resolved by discussions with the third reviewer. IK imported the extracted data into the summary table for systematic review and two reviewers (JK and ZRC) verified it. JK imported the extracted data and prepared it for meta-analysis and two reviewers (ZRC and IK) verified it. Biostatistician (AL) reviewed and confirmed the final data for meta-analysis. All co-authors reviewed the final tables and figures. This systematic review and meta-analysis followed the PRISMA DTA guidelines^41^ and the study protocol was registered with PROSPERO, CRD42023391560.

### Data analysis

We extracted key information, including true positive, false positive, false negative, and true negative information among clinicians with and without AI assistance. We generated contingency tables, where possible, to estimate diagnostic test accuracy in terms of pooled sensitivity and specificity. Additional information about the AI algorithm (e.g., architecture, image sources, validation and AI assistance method), participants, patients, target condition, reference standard, study setting and design, and funding were extracted.

A revised tool for the methodological quality assessment of diagnostic accuracy studies (QUADAS-2)^42^ was used to assess risk of bias and concerns of applicability of each study in four domains, patient selection, index test, reference standard, and flow and timing (Supplementary Table 1). A pair of reviewers independently evaluated the domains, compared the ratings, and, if conflicted, reconciled the discrepancies through discussions led by the third reviewer (IK, JK, ZRC).

We used the Metandi package^43^ for Stata 17 (College Station, TX) to compute summary estimates of sensitivity and specificity with 95% confidence intervals (95% CI) of humans with AI-assistance compared to humans without AI assistance using a bivariate model^44^. Summary Receiver Operating Characteristics (SROC) curves were plotted to visually present the summary estimates of sensitivity and specificity with 95% confidence region and the 95% prediction region, which refers to the confidence areas that the sensitivity and specificity of future studies likely fall into. The Bivariate models were performed separately for clinicians with vs. without AI assistance because the Metandi package could not handle the paired design of the data. We applied a random effects model to account for the anticipated heterogeneity across studies, potentially due to the variance of the data, including the use of different AI algorithms, medical professionals, and study settings. Heterogeneity was assessed by visual inspection of graphics, including SROC curve and forest plots^45,46^. Additionally, we conducted bivariate meta-regression analysis using the Meqrlogit package (Stata 17, College Station, TX) by the presence of AI assistance or not, for each experience level in dermatology (dermatologists, residents, non-dermatology medical professionals), type of diagnostic task (binary classification or top diagnosis) and type of image (clinical or dermoscopic) separately, to compare diagnostic accuracy by AI assistance and adjust for the potential heterogeneity caused by these factors^47^. To investigate the presence of a positive threshold effect, Spearman correlation coefficient was computed between sensitivity and specificity^48^. Pre-planned sensitivity analyses were conducted by excluding potential outliers,^49^ studies with poor methodology (where at least three domains were rated as unclear or high bias), and studies with reference standards other than only histopathology. We examined publication bias using Deeks’ Funnel Plot Asymmetry Test, which ran a regression on the effective sample size funnel plots vs. diagnostic odds ratio^50^. We calculated κ statistics to evaluate the agreements between QUADAS-2 assessors. All statistical significance was determined at *p* < 0.05.

### Supplementary information

Supplementary Information

## Data Availability

E.L. has full access to all the data in the study and takes responsibility for the integrity of the data and the accuracy of the data analysis. All study materials are available from the corresponding author upon reasonable request.

## Code availability

The codes used in the analysis of this study will be made available from the corresponding author upon reasonable request.
