# Supplementary material for: Human-AI interaction in skin cancer diagnosis: a systematic review and meta-analysis
Source: NPJ Digit Med. 2024 Apr 9;7:78. doi: 10.1038/s41746-024-01031-w (PMC11004168; doi:10.1038/s41746-024-01031-w)
Supplement: Supplementary file 1 — Supplementary Information [file 41746_2024_1031_MOESM1_ESM.pdf]

Supplementary Material

Supplementary Table 1. Assessment of methodological quality of studies using QUADAS-2

	Risk of bias				Applicability concerns		
Study	Patient selection	Index test	Reference standard	Flow and timing	Patient selection	Index test	Reference standard
Cho 2020	Low	Low	Low	Low	Low	Low	Low
Han 2020	Low	Low	Low	low	Low	Low	Low
Han 2022	Low	Low	Low	Low	Low	Unclear	Low
Jahn 2022	High	Low	High	High	High	Unclear	High
Kim 2022	Low	Low	Low	Low	Low	Low	Low
Lee 2020	Low	Low	Low	Low	Low	Low	Low
Tschandl 2020	Low	Low	Low	Low	Low	Low	Low
Maron 2020	Low	Low	Low	Unclear	Low	Low	Low
Lucius 2020	Unclear	Low	Low	Low	Unclear	Low	Low
Ba 2022	Low	Low	Low	Low	Low	Low	Low
Jain 2021	Low	Low	Low	Low	Low	Low	Low
Muñoz-López 2021	Low	Low	Low	Low	Low	Low	Low

Supplementary Figure 1. Deek's Funnel Plot Asymmetry Test

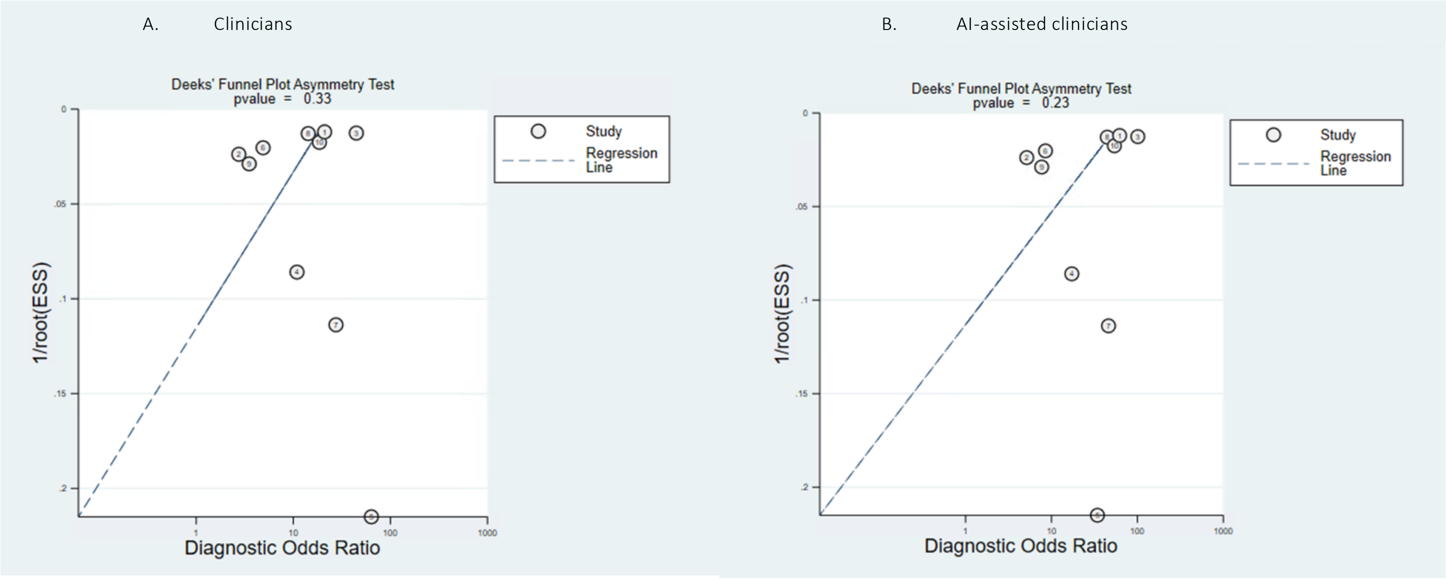

Supplementary Table 2. Sensitivity analyses

	Clinicians		AI-assisted clinicians	
Overall	SE	74.8% (68.6-80.1)	SE	81.1% (74.4-86.5)
	SP	81.5% (73.9-87.3)	SP	86.1% (79.2-90.9)
Without outliers	SE	78.8% (75.1-82.1)[1,2]	SE	83.7% (78.8-87.7)[1]
	SP	83.6% (78.0 – 88.1)[3]	SP	89.4% (86.6-91.7)[2,3]
Without low-quality study	SE	74.2% (68.0-79.6)[4]	SE	89.4% (86.6-91.7)[4]
	SP	80.2% (72.2-86.3)[4]	SP	85.9% (78.3-91.2)[4]
Without studies with reference standards other than only histopathology	SE	75.5 (56.3-88.0)[3,5-9]	SE	78.2 (62.9-88.3)[3,5-9]
	SP	78.6 (72.3-83.8)[3,5-9]	SP	83.6 (77.0-88.7)[3,5-9]

Abbreviations: SE: sensitivity, SP: specificity. Reference indicates the outliers, low-quality study or studies not only using histopathology as reference standard, that were excluded from the analyses. [1]Jain et al. 2021, DOI: 10.1001/jamanetworkopen.2021.7249. [2]Maron et al. 2020, DOI: 10.2196/18091, [3]Cho et al. 2020, DOI: 10.1111/bjd.18459, [4]Jahn et al. 2022, DOI: 10.3390/cancers14153829, [5]Han et al. 2022, DOI: 10.1016/j.jid.2022.02.003, [6]Han et al. 2020, DOI: 10.1016/j.jid.2020.01.019, [7]Kim et al. 2022, DOI: 10.1371/journal.pone.0260895, [8]Lee et al. 2020, DOI: 10.1111/jdv.16185, [9]Tschandl et al. 2020, DOI: 10.1038/s41591-020-0942-0

Supplementary Table 3: Search Strategy

Set #	Concept	Syntax	Results
1	Artificial Intelligence	"Artificial Intelligence" [mesh] OR "Neural Networks, Computer"[Mesh] OR "Artificial Intelligence" [tw] OR "Neural Network" [tw] OR "Neural Networks"[tw] OR "Deep Learning" [tw] OR "Machine Learning" [tw] OR “algorithm*”[tw] OR “augmented intelligence”[tw]	663,431
2	Skin cancer	"skin neoplasms"[MeSH Terms] OR "Melanoma"[Mesh] OR "Skin Diseases"[MeSH Terms:noexp] OR "skin neoplasms"[tw] OR "skin neoplasm"[tw] OR "skin cancer"[tw] OR "skin cancers"[tw] OR "cancer of the skin"[tw] OR melanoma[tw] OR melanomas[tw] OR “skin diseases”[tw] OR “skin disease”[tw] OR ((skin[tw]) AND (lesion[tw] OR lesions[tw] OR malignant[tw] OR malignancy[tw] OR malignancies[tw])) OR “skin tumor*”[tw] OR “cutaneous tumor*”[tw]	418,646
3	Diagnosis	"Diagnosis"[Mesh] OR "diagnosis" [Subheading] OR “Diagnostic Imaging"[Mesh] OR "diagnostic imaging" [Subheading] OR diagnos*[tw] OR detect*[tw] OR recognition[tw] OR classification[tw] OR classify[tw]	13,515,457
4	Doctors or dermatologists	"Physicians"[Mesh] OR physician*[tw] OR doctor*[tw] OR provider*[tw] OR dermatologist*[tw] OR Practitioner*[tw] OR clinician*[tw]	1,312,282
5	combining	#1 AND #2 AND #3 AND #4	862
6	Limit to published 2017 to present, exclude reviews, case reports, editorials	#5 AND 2017:3000[dp] NOT ("Review" [Publication Type] OR "Case Reports" [Publication Type] OR "Editorial" [Publication Type])	433

Embase Search Strategy:
Date searched: 11/8/22

Set #	Concept	Syntax	Results
1	Artificial Intelligence	'artificial intelligence'/exp OR 'machine learning'/exp OR 'Artificial Intelligence':ti,ab,kw OR 'Neural Network':ti,ab,kw OR 'Neural Networks':ti,ab,kw OR 'Deep Learning':ti,ab,kw OR 'Machine Learning':ti,ab,kw OR 'algorithm*':ti,ab,kw OR 'augmented intelligence':ti,ab,kw	747,280
2	Skin cancer	'skin tumor'/exp OR 'melanoma'/exp OR 'skin disease'/de OR 'skin neoplasms':ti,ab,kw OR 'skin neoplasm':ti,ab,kw OR 'skin cancer':ti,ab,kw OR 'skin cancers':ti,ab,kw OR 'cancer of the skin':ti,ab,kw OR melanoma:ti,ab,kw OR melanomas:ti,ab,kw OR 'skin diseases':ti,ab,kw OR 'skin disease':ti,ab,kw OR ((skin:ti,ab,kw) AND (lesion:ti,ab,kw OR lesions:ti,ab,kw OR malignant:ti,ab,kw	674,115

		OR malignancy:ti,ab,kw OR malignancies:ti,ab,kw)) OR 'skin tumor*':ti,ab,kw OR 'cutaneous tumor*':ti,ab,kw	
3	Diagnosis	'diagnosis'/exp OR 'diagnostic imaging'/exp OR 'diagnosis'/lnk OR diagnos*:ti,ab,kw OR detect*:ti,ab,kw OR recognition:ti,ab,kw OR classification:ti,ab,kw OR classify:ti,ab,kw	13,619,444
4	Doctors or dermatologists	'physician'/exp OR physician*:ti,ab,kw OR doctor*:ti,ab,kw OR provider*:ti,ab,kw OR dermatologist*:ti,ab,kw OR Practitioner*:ti,ab,kw OR clinician*:ti,ab,kw	2,120,134
5	combining	#1 AND #2 AND #3 AND #4	1,488
6	Limit to published 2017 to present, exclude reviews, case reports, editorials	#5 AND [2017-2022]/py NOT ('case report'/de OR 'editorial'/it OR 'review'/it)	825

Scopus Search Strategy:
Date searched: 11/08/22

Set #	Concept	Syntax	Results
1	Artificial Intelligence	TITLE-ABS-KEY("Artificial Intelligence" OR "Neural Network" OR "Neural Networks" OR "Deep Learning" OR "Machine Learning" OR "algorithm*" OR "augmented intelligence")	4,589,018
2	Skin cancer	TITLE-ABS-KEY ("skin neoplasms" OR "skin neoplasm" OR "skin cancer" OR "skin cancers" OR "cancer of the skin" OR melanoma OR melanomas OR “skin diseases” OR “skin disease” OR ((skin) AND (lesion OR lesions OR malignant OR malignancy OR malignancies)) OR “skin tumor*” OR “cutaneous tumor*”)	573,258
3	Diagnosis	TITLE-ABS-KEY (diagnos* OR detect* OR recognition OR classification OR classify)	12,881,019
4	Doctors or dermatologists	TITLE-ABS-KEY (physician* OR doctor* OR provider* OR dermatologist* OR Practitioner* OR clinician*)	2,044,478
5	combining	#1 AND #2 AND #3 AND #4	1,733
6	Limit to published 2017 to present, exclude reviews, case reports, editorials	#5 AND (LIMIT-TO (PUBYEAR , 2023) OR LIMIT-TO (PUBYEAR , 2022) OR LIMIT-TO (PUBYEAR , 2021) OR LIMIT-TO (PUBYEAR , 2020) OR LIMIT-TO (PUBYEAR , 2019) OR LIMIT-TO (PUBYEAR , 2018) OR LIMIT-TO (PUBYEAR , 2017)) AND (EXCLUDE (DOCTYPE , "re") OR EXCLUDE (DOCTYPE , "ed"))	1,030

IEEE Search Strategy:
Date searched: 11/08/22

Set #	Concept	Syntax	Results
1	Artificial Intelligence	"Artificial Intelligence" OR "Neural Network" OR "Neural Networks" OR "Deep Learning" OR "Machine Learning" OR "algorithm*" OR "augmented intelligence"	1,440,890
2	Skin cancer	"skin neoplasms" OR "skin neoplasm" OR "skin cancer" OR "skin cancers" OR "cancer of the skin" OR melanoma OR melanomas OR “skin diseases” OR “skin disease” OR “skin tumor*” OR “cutaneous tumor*” OR lesion OR lesions OR malignant OR malignancy OR malignancies	16,357
3	Diagnosis	diagnos* OR detect* OR recognition OR classification OR classify	1,124,080
4	Doctors or dermatologists	physician* OR doctor* OR provider* OR dermatologist* OR Practitioner* OR clinician*	87,163
5	combining	#1 AND #2 AND #3 AND #4	963
6	Limit to published 2017 to present, exclude reviews, case reports, editorials	#5 AND limit to 2017-2022	687
